# Effect of Low-Concentration Hydrofluoric Acid Etching on Shear Bond Strength and Biaxial Flexural Strength after Thermocycling

**DOI:** 10.3390/ma13061409

**Published:** 2020-03-20

**Authors:** You-Jung Kang, Yooseok Shin, Jee-Hwan Kim

**Affiliations:** 1 Department of Prosthodontics, Oral Science Research Center, College of Dentistry, Yonsei University, Seoul 03722, Korea; kyj1219@yuhs.ac; 2 Department of Conservative Dentistry, Oral Science Research Center, College of Dentistry, Yonsei University, Seoul 03722, Korea; densys@yuhs.ac

**Keywords:** hydrofluoric acid, hot etching, shear bond strength, flexural strength, zirconia

## Abstract

This study evaluated the shear bond strength (SBS) and biaxial flexural strength (BFS) of resin cements according to the surface treatment method using low-temperature hot etching with hydrofluoric acid (HF) on a yttrium-stabilized tetragonal zirconia (Y-TZP) surface; 96 discs and 72 cubes for BFS and SBS tests for Y-TZP were randomly divided into four groups of BFS and three groups of SBS. Specimens were subjected to the following surface treatments: (1) no treatment (C), (2) air abrasion with 50 μm Al_2_O_3_ particles (A), (3) hot etching with HF at 100 °C for 10 min (E), and (4) air abrasion + hot etching (AE). After treatments, the specimens were coated with primer, and resin cement was applied with molds. The specimens were evaluated for roughness (Ra) via scanning electron microscopy and x-ray diffraction, and the data were analyzed by an analysis of variance (ANOVA) and Kruskal–Wallis tests. Group E produced significantly higher SBS compared to group A and AE before and after thermocycling. The BFSs of all groups showed no significant differences before thermocycling; however, after thermocycling, C and E treatment groups were significantly higher compared to group A and AE. All groups showed phase transformation. Group E was observed lower monoclinic phase transformation compared to other groups.

## 1. Introduction

The use of partially stabilized zirconium dioxide ceramics in dental restorations has recently been increasing owing to their excellent physical properties, such as high hardness, high compressive strength, optimal biocompatibility in a variety of applications, and adequate optical properties [1,2]. In particular, due to the development of computer-aided design and manufacturing (CAD/CAM) technology, these ceramics are increasingly being used as prosthetic restoration materials [3]. 

Zirconia restorations are not silica-based ceramics; therefore, resin bonding is difficult. Numerous researchers have focused on characterizing the effects of zirconia surface treatment methods (STMs) on the adhesions between resin cement and zirconia [4,5,6,7]. STMs include the following five micro-mechanical and chemical bonding techniques: mechanical treatment, chemical treatment, lasers, silicon coatings, and coupling agents [8]. 

Among the mechanical treatment techniques, the most commonly used is airborne-particle abrasion. Several studies have found that the abrasion method contributes to the increase in the adhesive strength between the resin cement and zirconia. However, the effects of the surface treatment vary according to the specific type of abrasion method; in particular, there are uncertainties regarding the impact of its long-term use [9,10]. For example, airborne-particle abrasion using alumina particles (Al_2_O_3_), the positive effects of which have been evaluated and reported by several studies using meta-analysis [11,12,13,14,15,16], eliminates impurities, induces surface modification, and increases surface roughness (known as Ra) on yttria-stabilized zirconia surfaces (Y-TZP). The resulting treatment features a rigid, durable micro-mechanical retention between the Y-TZP and resin cement, with significantly increased flexural strengths of the material; however, this method also induces the phase transformation of the zirconia surface crystal structure from tetragonal to monoclinic. The formation of micro-cracks due to this transformation can negatively affect the long-term stability of the zirconia [17,18,19,20]. In addition, airborne-particle abrasion is influenced by the particle size of the alumina, the pressure and distance at which air is applied, and the uniformity of abrasion on the surface; this is especially true in those cases in which excessive particle size and reduced application distance induce micro-crack formation. This is a disadvantage which not only reduces the long-term mechanical properties of the ceramic, but also makes it difficult to apply uniform surface treatment within dental clinics [17,18,21,22]. Due to these challenges, new methods of zirconia surface modification, such as surface coating, laser irradiation, and acid etching, including a form of chemical etching known as “selective infiltration etching” (SIE), are being developed to improve the resin’s adhesion [23,24,25,26]. In particular, there have been studies that use chemical etching via different approaches to increase the adhesive strength [8,27].

Zirconia was originally known as a material that is difficult to etch using hydrofluoric acid (HF) solution [28,29]; however, it has now been shown that better adhesion to zirconia can be attained by changing the formulation and application of the etching solution [4,19,23,30,31,32], thus improving the etching conditions. Previous studies have used various etching solutions of high to low concentrations, including HF, hydrochloric acid (HCl), sulfuric acid (H_2_SO_4_), and nitric acid (HNO_3_) [4,23,31,33]. Lui et al. compared the shear bond strength (SBS) of the resin–zirconia bond after various surface treatments including those using high concentrations (above 9.5%) of HNO_3_ and HF [33]. The surface treatment involving the use of high-concentration HF solutions for 25 min at 100 °C and silica coating is effective in forming a micro-retentive structure and has been reported to improve the reliability of the resin–zirconia bonding without phase transformation from tetragonal to monoclinic.

In a previous study [28], the SBS between zirconia and resin cement did not show significant improvement for treated zirconia with a low concentration of 9.5% HF. Other previous studies using 9.5% HF [34] confirmed that the production of porosities as micro-morphological changes increased according to the time and temperature by comparing the 1-min treatment at 80 °C to 1-h treatment at 25 °C. 

Some studies have found that hot chemical etching solutions can produce a significantly better Ra than that obtained with the SIE methods and that hot etching can improve the mechanical retention of zirconia and increase the mean arithmetic profile deviation roughness parameter (Ra_mean_) [4,30]. For hot etching, the etching effect was increased and the adhesion between the zirconia and resin was improved when heat was applied to the surface treatment of zirconia using the etching solution [23,30,31,32]. It has also been demonstrated that hot etching may result in a selective chemical etching of zirconia, which leads to the production of micro retention and increases the grain boundaries through the preferential removal of the less-arranged and high-energy peripheral atoms [32]. However, using high-concentration etching solutions in a clinic at high temperatures would be dangerous.

Although HF at concentrations of 4%–10% was reported to be safe for dental use [35], few studies have been conducted on SBS and biaxial flexural strength (BFS) when hot etching is performed for a short time with low-concentration HF at high temperatures. There has also been very little research on characterizing the properties of zirconia strength and surface changes after thermocycling. Therefore, the development of a surface treatment that is safer for use in clinics while maintaining the integrity of the adhesive strength would appear to be necessary.

To address this need, our study aims to evaluate the effect of hot etching surface treatment using low-concentration HF (9%) at a low temperature on the SBS between partially stabilized zirconia ceramic and resin cement and the BFS of zirconia.

## 2. Materials and Methods

### 2.1. Sample Preparation

The primary material used for this study was pre-sintered yttria-tetragonal zirconia polycrystal (Dmax Natura Z-W9812, DMAX Co., Daegu, Korea), which was converted from block form into discs using a CAD/CAM process.

For the BFS test, the discs were sintered at 1530 °C for 2 h (heating rate: 3 h from 20 °C to 900 °C, and 4 h from 900 °C to 1530 °C, followed by natural cooling); subsequently, these were polished with a 1-µm diamond paste to produce the final dimensions of a diameter of 13.6 mm and a thickness of 1.5 mm, yielding a total of 96 zirconia specimens (n = 96).

For the SBS preparation, zirconia block cubes (dimensions 5 × 5 × 5 mm) were fabricated using the same sintering method and were placed with the adhesive side down; the resin was then poured into the mold (25 mm diameter by 15 mm high), followed by self-curing for 24 h. The upper surface of each specimen was polished with silicon–carbon abrasive papers (grits #400, 600, and 1200); this was achieved by using a polishing machine under tap water irrigation to remove contaminants. 

All specimens were then divided into four experimental study groups of 24 specimens each as follows: group C (control group), group A (abrasion group), group E (etching group), and group AE (abrasion-etching group). The specimens were then sonicated in water for 5 min according to the type of the surface treatment performed, as follows. 

**Figure 1:**
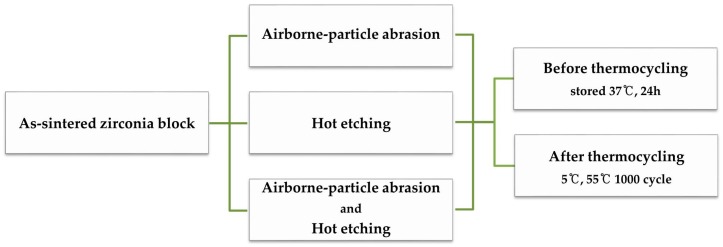
Specimen surface-treatment procedure.

For C, no further treatment was applied; this group served as a control. For A, the zirconia surfaces were airborne-particle-abraded with 50-µm aluminum oxide (Al_2_O_3_) particles (Cobra^®^ 50 µm, white; Renfert GmbH, Hilzingen, Germany) applied perpendicular to the surface at 0.2 MPa for 15 s at a distance of 10 mm. For E, after applying HF (Ceramic Etchant 9, Medifive Co., Ltd., Incheon, Korea) to the zirconia surfaces, the group was placed in a closed container that consisted of a triple-locking design with a heat-generating pack for 10 min. To neutralize the F ions and toxic substances emitted from the upper part of the closed vessel, a HF neutralizing agent was used (HF Neutralizer, Medifive Co., Ltd., Incheon, Korea). For AE, both airborne-particle abrasion and experimental hot etching solution treatments were applied. After surface treatment using the same airborne-particle method as that applied to group A, and water rinsing with ultrasound, and drying with an air syringe, the abraded zirconia surfaces were hot-acid-etched using the same method as that applied to group E. All the specimens were rinsed in an ultrasonic bath containing water for 5 min to remove the alumina particles or HF residue, and then dried using the air-water syringe. The specimen surface-treatment procedure is illustrated in Figure 1 and the materials are suggested in Table 1. Groups C, A, E, and AE were included in the BFS test and Groups A, E, and AE were included in the SBS test.

The surface-treated zirconia specimens were then coated with primer (Z-Prime Plus; BISCO, Schaumburg, Illinois, USA), as directed by the manufacturer and dried with an air-water syringe for 5 s. The resin–zirconia bonding method was as follows. Resin cement (TheraCem, BISCO, Schaumburg, Illinois, USA) was applied to a plastic mold (Ultradent Jig; Ultradent Products, South Jordan, USA), which was then placed on the zirconia surface (bonding area 4.45 mm^2^) and light-cured with a 1200 mW LED light curing unit (DB-686 Cappu LED Curing Light; Bisco Asia, Seoul, Korea). 

Finally, half of the specimens from each group (C, A, E and AE) were stored in distilled water at 37 °C for 24 h, and the other half of the specimens were thermally cycled for 1000 cycles between 5 °C and 55 °C with a dwell time of 10 s.

### 2.2. Shear Bond Strength (SBS) Test

For the evaluation of SBS (n = 12), each specimen was fixed in a universal testing machine (Bisco, Schaumburg, IL, USA) using a metal jig. The maximum force load (recorded in N) was measured at a crosshead speed of 0.5 mm/min at a distance of 1 mm from the bonding interface until the cement column was dropped. The bond strength for each specimen was calculated by dividing the peak load (in N) by the surface area (4.45 mm^2^) to achieve the strength in MPa.

### 2.3. Biaxial Flexural Strength (BFS) Test

The BFS (n = 12) tests were performed using the piston-on-three-ball technique in a universal testing machine (3366 Series, Instron Engineering, Norwood, MA, USA), which features three stainless-steel balls placed equidistant from each other on a support circle with a diameter of 9 mm. The discs were placed centrally facing the steel balls. A thin plastic sheet (0.05 mm thick) was positioned between the piston and the specimen to facilitate an even load distribution. The load was applied with a 1.5-mm diameter piston at a crosshead speed of 1.0 mm/min. The BFS of each specimen was calculated using the following equation obtained from the International Organization for Standardization (ISO) Standard 6872 with Poisson’s ratio value for dental ceramic as 0.25:

σ = −0.2387P (X − Y)/d^2^ (1)

where σ is the biaxial flexural strength (MPa), P is the total load causing the fracture (N), and d is the specimen disk thickness at the fracture origin (mm). X and Y were calculated as follows:

(2) X=(1+υ)ln(r2 / r3)2+[(1−υ) / 2](r2 / r3)2

(3) Y=(1+υ)[1+ln(r1 / r3)2]+(1−υ)(r1 / r3)2

where v is Poisson’s ratio (υ), r1 is the radius of the support circle (4.5 mm), r2 is the radius of the loaded area (0.75 mm), and r3 is the radius of the specimen (6.8 mm).

### 2.4. Scanning Electron Microscopy (SEM)

To evaluate the difference between treatments, the surface morphology of each group (groups A, E, and AE before and after thermocycling, n = 1 per group) was observed with a field emission scanning electron microscope (JEOL-7800F Schottky, JEOL, Tokyo, Japan). The analysis procedures were performed after gold sputtering (Cressington High Resolution Sputter Coater 208HR, Cressington Scientific Instruments Ltd., Watford, UK) with a 40,000× magnification.

### 2.5. D Optical Microscopy

For the topographic analysis and determination of the Ra of the zirconia surfaces after different methods of treatment and thermocycling, six specimens from each group were analyzed in this study (Groups A, E, and AE before and after thermocycling, n = 1 per group) using a 3D optical microscope (Contour GT-X3 BASE; Bruker Co., Bremen, Germany), in five areas per sample. Subsequently, the average Ra values were calculated.

### 2.6. X-Ray Diffraction (XRD)

The percentages of monoclinic phase in each group (groups C, A, E, and AE both before and after thermocycling (n = 3 per group)) were calculated by high-resolution X-ray diffraction (HR-XRD; SmartLab, Rigaku, Tokyo, Japan). Scans were performed at 45 kV and 200 mA, from 25° to 36° with a 0.02° step size. The XRD patterns were analyzed using the Rietveld refinement methods; quantitative analyses by the reference intensity ratio method were performed using the PDXL software (PDXL V1.8.1.0, Rigaku, Tokyo, Japan). The monoclinic peak intensity ratio ($X_{m}$) was calculated using the equation reported by Garvie and Nicholsone [36], as follows:

(4) $$X_{m}=\frac{I_{m}\left(\bar{1}11\right)+I_{m}\left(111\right)}{I_{m}\left(\bar{1}11\right)+I_{m}\left(111\right)+I_{t}\left(111\right)}$$

where $I_{t}$ and $I_{m}$ represent the integrated intensities of tetragonal (111)_t_ peak and monoclinic (111)_m_ and (−111)_m_ peaks around 2θ = 30.2°, 31°, and 28.2°, respectively. The monoclinic phase content ($F_{m}$) was calculated using the equation reported by Taraya et al. [37], as follows:

(5) $$F_{m}=\frac{1.311X_{m}}{1+0.311X_{m}}$$

### 2.7. Statistical Analysis

The final results were analyzed using statistical software (SPSS 25.0, SPSS Inc., Chicago, IL, USA) by separately observing the resulting SBS and BFS values of the groups. First, the data normality was tested using the Kolmogorov–Smirnov and Shapiro–Wilk tests (with α = 0.05). The differences between SBS (MPa) and Ra values of the tested groups were analyzed using a one-way analysis of variance (ANOVA), which was followed by a t-test and Tukey’s honestly significant difference (HSD) test (*p* < 0.05). The BFS data (MPa) were analyzed using the variance analysis provided by the Kruskal–Wallis and Mann–Whitney U tests (*p* < 0.05).

## 3. Results

### 3.1. Shear Bond Strength (SBS)

The mean bond strength values (MPa ± SD) of the tested groups (A, E, and AE before and after thermocycling) are summarized in Table 2. The one-way ANOVA test showed a statistical difference in each surface treatment group (*p* < 0.05). With regard to the groups observed before and after thermocycling, group E demonstrated a significantly higher SBS than those of the other two groups. There were no significant differences between the SBS values of A and AE. In all the groups, the adhesive strength after thermal cycling was significantly lower than that observed before cycling.

### 3.2. Biaxial Flexural Strength (BFS)

The mean flexural strength values (MPa ± SD) for each group (C, A, E, and AE before and after thermocycling) are listed in Table 3. There was no significant difference in the BFS values of the three groups before thermocycling (*p* > 0.05). The Kruskal–Wallis test revealed a statistical difference in the thermocycling groups in each surface treatment group (*p* < 0.05). Groups C and E exhibit significantly higher BFS values than those of groups A and AE. There was no statistical difference between the BFS values of thermocycling groups C and E.

### 3.3. X-Ray Diffraction (XRD)

**Figure 2:**
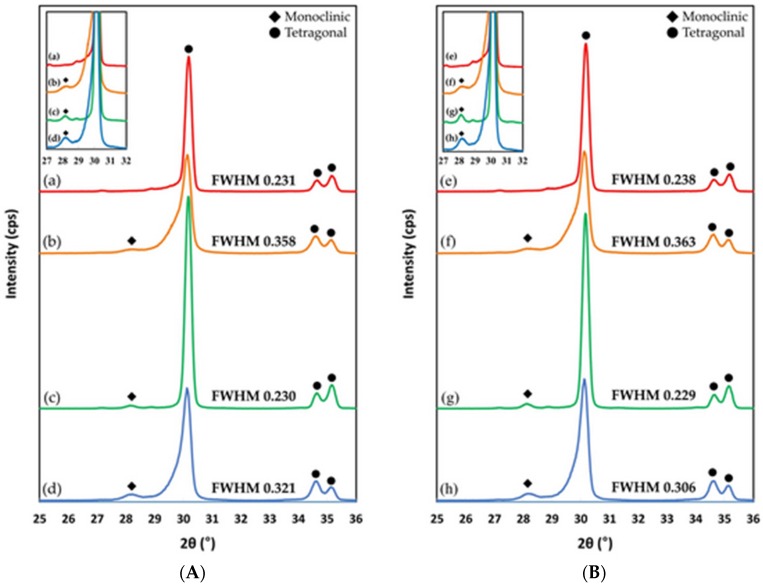
X-ray diffraction (XRD) pattern (**A**) before thermocycling of (a) group C, (b) group A, (c) group E, and (d) group AE. The XRD pattern (**B**) after the thermocycling of (e) group C, (f) group A, (g) group E, and (h) group AE. Both graphs show a difference in the monoclinic phase among the groups and contain the full width at half maximum (FWHM) of each group. The inset shows the magnified region.

Representative XRD patterns obtained from the eight groups are presented in Figure 2. Table 4 reports the mean monoclinic phase content values with standard deviation of the zirconia in the groups before and after thermocycling. The monoclinic phase structure could be detected on the zirconia surface in all the groups, and the monoclinic (−111) peak was detected at a 2θ of 28.2°. The monoclinic crystal phase in group E was the lowest and the corresponding value in group AE was higher than those achieved in other groups; this was observed before and even after thermocycling. In all groups, the tetragonal (111) peak at a 2θ of 30.2° was detected. In group E, the tetragonal peak was narrow both before and after thermocycling, and the respective full width at half maximum (FWHM) was 0.230 and 0.229. In group A, the tetragonal (111) peak was broadened and the FWHM of the peak increased from 0.231 to 0.358 before thermocycling; furthermore, the monoclinic content and FWHM increased up to 8.4% and 0.363, respectively, after thermocycling. Moreover, after thermocycling, the monoclinic phase peaks were observed to slightly increase in all the groups. The monoclinic phase content and FWHM of group AE slightly decreased after thermocycling. However, the monoclinic phase content was still higher than in other groups. However, in group E, the monoclinic phase content was lower than those of groups A and AE.

### 3.4. Scanning Electron Microscopy (SEM)

**Figure 3:**
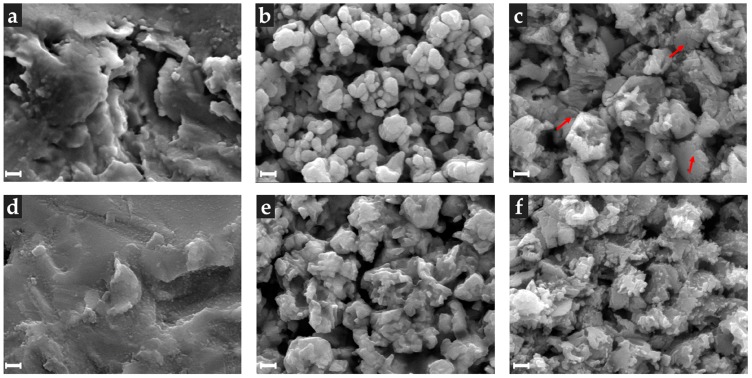
Scanning electron microscopy photomicrographs of zirconia ceramic disc after different surface treatments: zirconia stored in distilled water at 37 °C for 24 h ((**a**), (**b**), and (**c**)) and thermocyced zirconia (1000 cycles between 5 °C and 55 °C ((**d**), (**e**) and (**f**))); (**a**,**d**) airborne-particle abrasion (group A); (**b**,**e**) hydrofluoric acid (HF) etching (heated up to 100 °C, 10 min) (group E); (**c**,**f**) HF acid etching after airborne-particle abrasion (heated up to 100 °C, 10 min) (group AE). (Scale: 100 nm; Magnification: 40 k).

Figure 3 shows the morphological appearance of zirconia surface after being modified with the various methods used in this study. For group A, the zirconia appeared to have deep and rough abrasions. For E, an overall homogenous and fine rough surface was observed, while for AE, the deep areas due to abrasion formed fine rough surfaces, which was probably attributable to the hot etching. For each of the thermocycling groups A, E, and AE, the surfaces had smooth edges. It was also noted that the AE treatment resulted in signs of micro-crack formation. 

### 3.5. Surface Roughness (Ra)

**Figure 4:**
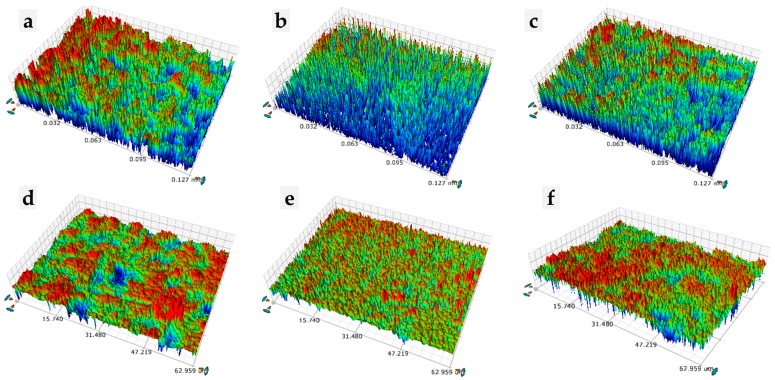
Roughness (Ra) images of a zirconia ceramic disc after different surface treatments: zirconia stored in distilled water at 37 °C for 24 h ((**a**), (**b**), and (**c**)) and those of thermocycled zirconia (1000 cycles between 5 °C and 55 °C ((**d**), (**e**), and (**f**))); (**a**,**d**) airborne-particle abrasion (group A); (**b**,**e**) HF acid etching (heated up to 100 °C, 10 min) (group E); (**c**,**f**) HF acid etching after airborne-particle abrasion (heated up to 100 °C, 10 min) (group AE).

Figure 4 shows the outcomes of Ra after the initial surface treatment of each group, and after thermocycling following the initial treatment. Table 5 reports the mean Ra values with the standard deviation of the zirconia in the groups before and after thermocycling.

## 4. Discussion

This study investigated the effect of hot chemical etching on the adhesive strength and fracture strength between zirconia and a resin cement after thermocycling; the etching was carried out with HF at a low concentration of 9%. As a result, the comparison between the average adhesive strength and the surface treatment methods revealed that not only was the adhesive strength of the HF etching group significantly higher than that of the control group using airborne-particle abrasion, but also that the fracture strength did not decrease significantly.

Surface treatment methods for improving the adhesion strength of zirconia have been classified into the utilization of mechanical and chemical treatments, laser irradiation, silicon coatings, and coupling agents [8,12,23,33,38,39]. In particular, there have been numerous studies on methods using airborne-particle abrasion, which is a mechanical treatment that increases the roughness of the zirconia surface and results in high bond strengths when defects occur with the phosphate ester monomers [33,39]. However, airborne-particle abrasion may be affected by variables such as particle size, injection distance, time, and pressure [17,40]. Depending on the size of the particles, a substantial strength degradation can be observed, and under the application of high pressures [14], the surface roughness may be improved; however, this could also lead to a reduction in the bond strength. Furthermore, it has been reported that the bonding capability of resin cement to zirconia substrate is significantly influenced by the method of airborne-particle abrasion and low reliability for long-term use [32]. 

HF acid is one of the most commonly used materials in chemical treatment methods for etching dental ceramics containing silica. However, zirconia with treated HF solution does not induce a surface change because it is a glass-free material; therefore, zirconia cannot bond with the resin cement [27,41]. Recently, zirconia surface treatments for various concentrations of HF, temperatures, and application times have been studied. Temperature plays a very important role in molecular motion, as hot etching renders the proton of the acid solution prone to ionization; therefore, it becomes even more acidic, thereby accelerating the removal of surface particles [4,23,31,32,33]. Smielak et al. [19] reported that zirconia surface roughness has a positive effect on the quality of the surface as the etching time and HF concentration increase. However, a high temperature and high concentration etching solution is not safe for use in dental clinics or laboratories and is difficult to handle. Typically, HF with a concentration of 4%–10% is used in a dental clinic, and this concentration range has been reported to be safe for dental use [35]. Therefore, in the present study, surface analysis and strength tests were performed after heat treatment using low-concentration HF (9%) and by employing triple-sealed containers.

Our results show a micro-morphological change in the surface and homogeneous roughness due to the hot etching with a low concentration of HF (Figure 2). The airborne-particle abrasion group showed a rough and irregular surface, whereas the hot etching group showed that the space between the grains was increased due to the decrease in particle size and the uniform application of the treatment. These results are in agreement with Sriamporn et al. [34].

In the present study, phase transformation occurred before and after the thermocycling for all groups subjected to the surface treatment, which is similar to previous studies; this suggests that airborne-particle abrasion and etching can lead to a phase transformation [7,34,42]. In particular, the increased monoclinic phase before and after thermocycling in the etching surface treatment can be presumed to be due to the structural deformation caused by heat treatment during etching. In this study, heat treatment with steam was carried out using an airtight container with a triple structure during the etching process. Wet heat treatment using low-temperature degradation (LTD) (room temperature to 400 °C) on zirconia can transform partially stabilized zirconia from the tetragonal to the monoclinic phase. However, in this study the integrity of the zirconia surface treated with hot etching did not decrease; therefore, it is considered that the phase transformation by hot etching does not degrade the mechanical properties.

The Ra value of zirconia in this study was lower in the etching group than in the abrasion group (Table 4). It is speculated that this result could be due to the formation of rough structures and cracks caused by uneven and concentrated pressure during the abrasion surface treatment, and the formation of the nano-porosity structure that is uniformly treated during the hot etching surface treatment. In previous studies [5], a lower Ra value than that observed in the abrasion group was measured in the etching surface treatment; however, the SBS value before and after thermocycling was significantly higher than that of the abrasion group. These results are in agreement with the present study, which suggests that although the average surface roughness was improved when treated with airborne-particle abrasion, hot etching is more appropriate for zirconia and cement bonding.

The present study also demonstrated that the hot etching treatment group significantly enhanced the SBS of resin cement to zirconia before and after thermocycling (*p* < 0.05). Previous studies have reported that bonding of nano-porosity and resin on HF-treated zirconia surfaces is difficult [34]. However, the hot etching group showed the highest SBS values before and after thermocycling in another study [5,32]. In the present study, the SBS value was significantly lower in all groups after thermocycling than that observed before it. Furthermore, after thermocycling, the hot etching group of the three groups showed significantly higher SBS values. It could be speculated that the hot etching treatment contributed to a micro-locking formation between the uniformed nano-structure of zirconia and the resin cement, which may have resulted in an enhanced bond strength by the mechanical bonding effect of MDP contained in the resin cement. Due to the high bond strength observed after the hot etching process, it can be speculated that, when the resin penetrates the surface of the zirconia, it could be more readily structurally bonded with the etched surface.

In the present study, there was no difference in the BFS between all the groups before thermocycling. However, the average BFS value of the group subjected to hot etching treatment is significantly higher than that of the other treated groups after thermocycling (*p* < 0.05). Although studies on achieving an increased strength in etched zirconia due to thermocycling are limited, the results of this study demonstrate that the BFS of the etched zirconia did not degrade even after thermocycling.

## 5. Conclusions

Within the limitations of this study, it was concluded that hot etch surface treatment with low concentrations of HF had a higher shear bond strength and flexural strength than other groups before and after thermal cycling, which had a positive effect on the surface treatment. However, further in-vitro studies concerning the long-term thermocycling and a large number of specimen studies will be necessary to thoroughly evaluate the effects of the hot etching surface treatment with low concentrations of HF. These studies will complement the hot etching methods that are available in clinics.

## Figures and Tables

**Table 1:** Materials used in this study.

Material	Product Name	Main Composition ^a^	Manufacturer
Zirconia block	D max Natura Z-W9812	<95% ZrO_2_ + HfO_2_ <6% Y_2_O_3_	DMAX Co., Daegu, Korea
Ceramic Primer	Z-Prime Primer	<90% Ethanol <10% Biphenyl dimethacrylate <20% 2-hydroxyethyl methacrylate (HEMA), <5% MDP	Bisco, Inc. Schaumburg, USA
Self-adhesive resin cement	TheraCem^®^	Base <50% Portland cement <50% Ytterbium w/ Barium Glass <5% Ytterbium Fluoride <5% BisGMA Catalyst paste <30% 10-Methacryloyloxydecyl Dihydrogen Phosphate, <5% 2-Hydroxyethyl Methacrylate	
Hydrofluoric acid	Zirconia Etchant	9% Hydrofluoric acid gel <10% hydrofluoric acid <2% thickening agent	Medifive Co., Ltd., Korea

^a^ Main composition was accrued from safety data sheets provided by manufacturers; wt.%, weight percent.

**Table 2:** Adhesion assessed by shear bond strength (MPa) of different surface treatment groups (mean ± standard deviation).

Surface Treatment	Group A	Group E	Group AE
Before thermocycling	24.9 ± 3.0 ^a,A^	29.5 ± 2.9 ^b,A^	23.7 ± 3.1 ^a,A^
After thermocycling	11.4 ± 4.7 ^a,B^	15.5 ± 3.8 ^b,B^	11.5 ± 3.3 ^a,B^

^a,b^ Different superscript upper-case letters indicate groups that are statistically different within the same row between the surface treatment groups (*p* < 0.05). ^A,B^ Different superscript upper-case letters indicate groups that are statistically different within the same column between the surface treatment groups (*p* < 0.05).

**Table 3:** Biaxial flexural strength of different surface treatment groups (MPa).

Surface Treatment	Group C	Group A	Group E	Group AE
	Median (Q1–Q3)			
Before thermocycling	1356.7 ^a,A^ (1324.6–1439.4)	1412.8 ^a,A^ (1308.3–1452.3)	1415.6 ^a,A^ (1371.3–1459.7)	1416.0 ^a,A^ (1294.6–1538.7)
After Thermocycling	1289.0 ^a,A^ (1044.1–1355.0)	616.7 ^b,B^ (569.3–700.2)	1048.8 ^a,A^ (960.9–1455.6)	564.3 ^b,B^ (502.5–616.0)

^a,b^ Different superscript lower-case letters indicate groups that are statistically different within the same row between the treatment groups (*p* < 0.05). ^A,B^ Different superscript lower-case letters indicate groups that are statistically different within the same column between the treatment groups (*p* < 0.05).

**Table 4:** Monoclinic phase content ($F_{m}$.%) of zirconia with different surface treatment (Mean ± standard deviation).

Surface Treatment	Group C	Group A	Group E	Group AE
Before thermocycling	1.6 ± 0.1	7.8 ± 0.2	2.6 ± 0.2	10.3 ± 0.5
After thermocycling	1.7 ± 0.1	8.4 ± 0.1	4.2 ± 0.8	9.5 ± 0.2

**Table 5:** Surface roughness (Ra, μm) with different surface treatments. (Mean ± standard deviation).

Surface Treatment	Group A	Group E	Group AE
Before thermocycling	0.439 ± 0.02 ^a,A^	0.247 ± 0.04 ^b,A^	0.428 ± 0.02 ^a,A^
After thermocycling	0.355 ± 0.02 ^a,B^	0.181 ± 0.01 ^b,B^	0.353 ± 0.01 ^a,B^

^a,b^ Different superscript lower-case letters indicate groups that are statistically different within the same row between the surface-treatment groups (*p* < 0.05); ^A,B^ Different superscript upper-case letters indicate groups that are statistically different within the same column before and after thermocycling (*p* < 0.05 ).
